# Wavelength of light and photophobia in inherited retinal dystrophy

**DOI:** 10.1038/s41598-020-71707-2

**Published:** 2020-09-09

**Authors:** Yuki Otsuka, Akio Oishi, Manabu Miyata, Maho Oishi, Tomoko Hasegawa, Shogo Numa, Hanako Ohashi Ikeda, Akitaka Tsujikawa

**Affiliations:** 1 Department of Ophthalmology and Visual Sciences, Kyoto University Graduate School of Medicine, 54 Shogoin Kawara-cho, Sakyo-ku, Kyoto, 606-8507 Japan; 2 Department of Ophthalmology and Visual Sciences, Nagasaki University Graduate School of Biomedical Sciences, 1-7-1 Sakamoto, Nagasaki, 852-8501 Japan

**Keywords:** Eye diseases, Eye manifestations

## Abstract

Inherited retinal dystrophy (IRD) patients often experience photophobia. However, its mechanism has not been elucidated. This study aimed to investigate the main wavelength of light causing photophobia in IRD and difference among patients with different phenotypes. Forty-seven retinitis pigmentosa (RP) and 22 cone-rod dystrophy (CRD) patients were prospectively recruited. We designed two tinted glasses: short wavelength filtering (SWF) glasses and middle wavelength filtering (MWF) glasses. We classified photophobia into three types: (A) white out, (B) bright glare, and (C) ocular pain. Patients were asked to assign scores between one (not at all) and five (totally applicable) for each symptom with and without glasses. In patients with RP, photophobia was better relieved with SWF glasses {“white out” (p < 0.01) and “ocular pain” (p = 0.013)}. In CRD patients, there was no significant difference in the improvement wearing two glasses (p = 0.247–1.0). All RP patients who preferred MWF glasses had Bull’s eye maculopathy. Meanwhile, only 15% of patients who preferred SWF glasses had the finding (p < 0.001). Photophobia is primarily caused by short wavelength light in many patients with IRD. However, the wavelength responsible for photophobia vary depending on the disease and probably vary according to the pathological condition.

## Introduction

Inherited retinal degenerations (IRDs) represent a diverse group of diseases characterized by progressive photoreceptor cell death that can lead to blindness^1^. IRDs include many forms of retinal dystrophies such as retinitis pigmentosa (RP) and cone-rod dystrophy (CRD), with partially overlapping clinical and/or genetic findings. In addition to visual impairments such as nyctalopia, reduced visual acuity, and visual field defects, photophobia is a common symptom in patients with IRD^2^.

Photophobia is a common but often neglected symptom, even though it can severely impair a patient’s quality of life^3^. Many patients with IRD experience photophobia even in normal indoor illumination^4^. Tinted glasses may offer symptomatic relief but the most suitable lens color often varies among patients, indicating that the pathogenesis of photophobia is not exactly the same in each patient^5^. Nevertheless, considering that the symptom is induced by light stimulation and mitigated by tinted glasses, cells containing photopigments should be involved in the initial step of photophobia.

Photophobia is a sensory state in which light causes discomfort in the eye or head, but the exact definition and concept of photophobia are yet to be determined^6^. The details of the underlying mechanism of photophobia, such as the primary cells responsible for the symptom and the neural pathway of photophobia, are also controversial^5,7^. Nevertheless, it is generally believed that short-wavelength light plays a major role in photophobia, because it induces intraocular scattering^8^ and because of its potential energy^9^. Several previous reports have proposed the hypothesis that photophobia is primarily caused by cells that respond to short-middle wavelength light including S-cones^10^, intrinsically photosensitive retinal ganglion cells (ipRGCs)^5^, and rods^11^. The fact that patients with S-cone related diseases such as enhanced S-cone syndrome^12^, and blue cone monochromacy^8^ often complain of photophobia also supports the S-cone theory. However, patients with achromatopsia may complain photophobia, and the theory is still controversial. The mechanism may vary depending on the disease.

In this study, we investigated the wavelength of light primarily responsible for photophobia in patients with IRD. We also determined whether there is a difference between patients with different phenotypes.

## Methods

### Study subjects

This was a prospective observational study. We included consecutive patients with RP and CRD complaining of photophobia who visited Kyoto University Hospital from July 2018 to February 2019. All patients were clinically diagnosed with RP or CRD after comprehensive ocular examinations including indirect ophthalmoscopy, slit-lamp biomicroscopy, color fundus photography, and fundus autofluorescence (FAF) using a wide-field scanning laser ophthalmoscope (Optos, Optos PLC; Dunfermline, UK), spectral-domain optical coherence tomography (SD-OCT) (Spectralis; Heidelberg Engineering, Heidelberg, Germany), perimetry (Humphrey field analyzer; Carl Zeiss Meditec), and electroretinography (Neuropack MEB-2204; Nihon Kohden, Tokyo, Japan). Genetic testing was conducted on some patients using a previously described procedure^13^. We excluded patients who had other ocular diseases such as cystoid macular edema (on SD-OCT), corneal opacity, clinically significant cataract (Emmery-Little grade 3 or more, cortical opacity, and subcapsular opacity), or optic nerve disease that may be associated with photophobia. We also excluded patients who had undergone any eye surgery except for cataract surgery.

This study was approved by the ethics committee of the Kyoto University Graduate School of Medicine (Kyoto, Japan). All study participants gave written informed consent for this study. The research followed the tenets of the Declaration of Helsinki.

### Tinted glasses

**Figure 1:**
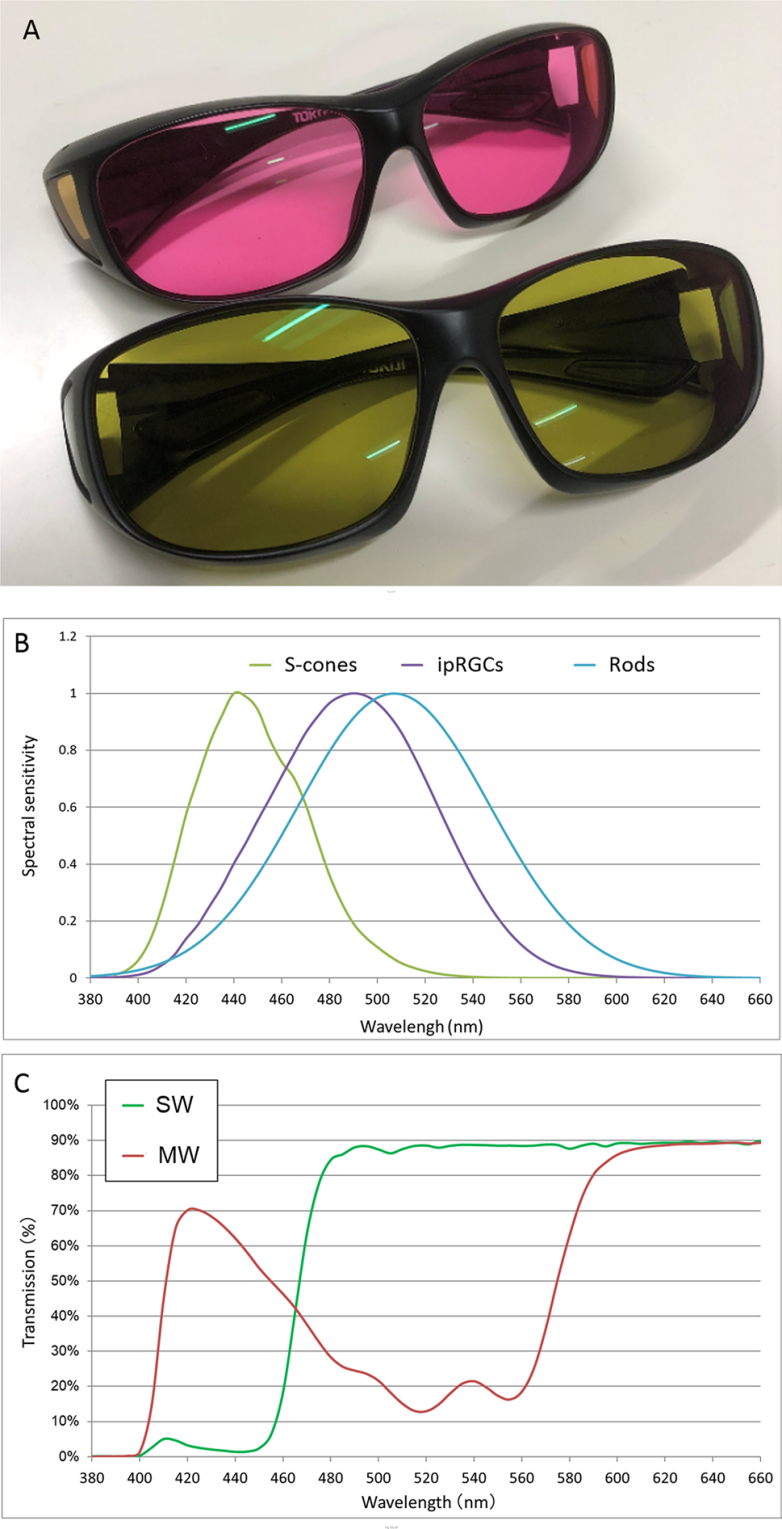
**(A)** The tinted glasses produced for the study. The green glasses filter short-wavelength light (SWF glasses). The other pink glasses filter middle-wavelength light (MWF glasses). **(B)** The spectral sensitivity curves of S-cones, intrinsic photosensitive retinal ganglion cells (iRGCs), and rods. Their sensitivity peaks at 430, 480, and 510 nm, respectively. **(C)** Spectral transmittance rate of each glasses. SWF glasses relatively restrict a transmission of short-wavelength light between 400 and 450 nm, targeting S-cones. MWF glasses restrict the range around 480–550 nm, targeting rods and ipRGCs.

We designed and produced two types of tinted glasses (Fig. 1A). The spectral sensitivity curves of S-cones, ipRGCs, and rods are shown in Fig. 1B. S-cones are potentially evoked at the peak of 430 nm (nm) light^14^. The action spectrum of iRGCs and rods peak at 480 nm^15^ and 500–510 nm^16^, respectively.

Green glasses (short wave filtering (SWF) glasses) were designed to restrict the transmission of short-wavelength light between 400 and 450 nm, targeting S-cones and attenuating their stimulation. The other pink glasses (middle wave filtering (MWF) glasses) were designed to restrict the range of light around 480–550 nm, targeting rods and ipRGCs (Fig. 1C). Spectral transmittance of the two glasses was measured between 350 and 800 nm using a spectrophotometer (U4100; Hitachi, Tokyo, Japan) with a one-nm spectral bandwidth. We confirmed that the total luminous transmittance was identical in two glasses.

### Questionnaire on the types and severity of photophobia

There are no established classifications or severity scales for the assessment of photophobia^6^. In the present study, we classified photophobia into three types according to the categories described in a previous study as follows^17^: (A) white out (a white out feeling on exposure to light), (B) bright glare (a symptom of bright glare on exposure to light), (C) ocular pain (ocular pain and feeling that a normal level of light is too intense)^18^. First, each patient looked at a white wall of a building in front of our hospital and the background landscape without glasses and was asked to assign a score from 1 to 5 (1. Not at all, 2. A little, 3. Neither, 4. Quite a lot, 5. Totally applicable) for each symptom. Next, each patient put on each pair of glasses in random order and the scores for the symptoms were recorded again. Patients were also asked which glasses relieved the symptoms more. To ensure that symptoms were detected to the maximum, all patients underwent this procedure with dilated pupils (0.5% phenylephrine and tropicamide) (SANDOL P Ophthalmic Solution; SANTEN, Osaka Japan); pupil dilation was also used for the routine clinical examination. The examinations were performed only on sunny days when the illumination intensity was more than 10,000 lx at eye level.

### Grading of Bull’s eye maculopathy

Presence or absence of Bull’s eye maculopathy was graded for each patient with RP. Bull’s eye maculopathy was defined as a ring-shaped atrophy of the outer retina and retinal pigment epithelium around the fovea. The grading was conducted by two independent graders (YO and AO) based on fundus photographs and FAF images. Agreement between the graders were evaluated.

### Data analyses

Data were expressed as mean ± standard deviation. T-tests and chi-square tests were used to compare values between groups. Visual acuity was measured with the Landolt chart and converted to the logarithm of minimum angle of resolution (logMAR) equivalent. All analyses were performed using a statistical analysis software (SPSS Statistics 19; SPSS, Inc., Chicago, IL, USA), and statistical significance was defined as p < 0.05.

## Results

Forty-seven patients with RP (21 men and 26 women) and 22 patients with CRD (14 men and 8 women) were included in this study; the mean ages of the patients were 55.2 ± 11.1 years and 57.8 ± 11.7 years, respectively. Causative genes including *EYS, USH2A, RP1, CNGA1, CNGB1, PRPF6, PRPF31, SNRNP20* was detected in 28 patients with RP (n = 15, 6, 2, 1, 1, 1, 1, 1, respectively), and *GUCY2D, PRPH2* were detected in two patients with CRD (n = 1, 1). Table 1 presents a summary of the comparison of the clinical characteristics of patients with RP and those of patients with CRD. As expected, the mean logMAR was significantly better in the RP group than in the CRD group (p < 0.01). In the RP group, approximately one-third of the patients (33.0%) had undergone cataract surgery, whereas only three patients in the CRD group (13.6%) had undergone the surgery. Blue-light-filter (yellow) intraocular lenses were implanted for all these patients.

**Table 1:** Clinical and ocular characteristics of patients with retinitis pigmentosa and cone-rod dystrophy.

	RP	CRD	p value
Number of patients	47	22	
Age	55.2 ± 11.1	57.8 ± 11.7	0.38
Sex (male/female)	21/26	14/8	0.14
log MAR	0.494 ± 0.601	0.993 ± 0.532	< 0.01
Phakia/IOL (eyes)	63/31	38/6	0.091

*RP* retinitis pigmentosa, *CRD* cone-rod dystrophy, *IOL* intraocular lens.

In both groups, the most severe symptom was “white out”. In the overall cohort, the types and severity of photophobia were measured without tinted glasses and reported in this order: white out, ocular pain, and bright glare (scores were 4.42 ± 0.88, 3.36 ± 1.29, and 2.04 ± 1.22, respectively). The average scores of photophobia improvement for each symptom in the RP and CRD groups are shown in Table 2. In the RP group, photophobia was better relieved with SWF glasses compared to MWF glasses, especially in patients with severe symptoms of “white out” (p < 0.01) and “ocular pain” (p = 0.013). Meanwhile, in the CRD group, there was no significant difference in the symptomatic relief felt by the patients after using the two glasses (p = 0.247–1.0). Most patients with RP (85.1%) preferred the SWF glasses, whereas approximately half of patients with CRD (54.5%) preferred the SWF glasses. The proportion of patients who preferred SWF glasses was significantly different between the two groups (p = 0.005).

**Table 2:** Improvement of photophobia severity score in patients with retinitis pigmentosa and cone-rod dystrophy with two types of tinted glasses.

		SWF glasses	MWF glasses	p value
**RP**	White out	2.68 ± 1.14	1.43 ± 1.12	< 0.01
	Bright glare	1.00 ± 1.12	0.87 ± 0.95	0.55
	Ocular pain	2.17 ± 1.31	1.51 ± 1.23	0.013
	Preferred glasses	40	7	
**CRD**	White out	2.36 ± 1.18	1.95 ± 1.13	0.247
	Bright glare	0.45 ± 0.96	0.45 ± 0.96	1
	Ocular pain	1.82 ± 1.18	1.50 ± 1.34	0.39
	Preferred glasses	12	10	

*RP* retinitis pigmentosa, *CRD* cone-rod dystrophy, *SWF glasses* short-wavelength light filtering glasses, *MWF glasses* middle-wavelength light filtering glasses.

In the RP group, 31.3% of patients who preferred SWF glasses had undergone cataract surgery, whereas 42.9% of those who preferred MWF glasses had done the surgery. There was no significant difference in the proportion (p = 0.547). In addition, the improvement in each symptom was not different between the phakic and pseudophakic patients (p = 0.193–0.957).

**Figure 2:**
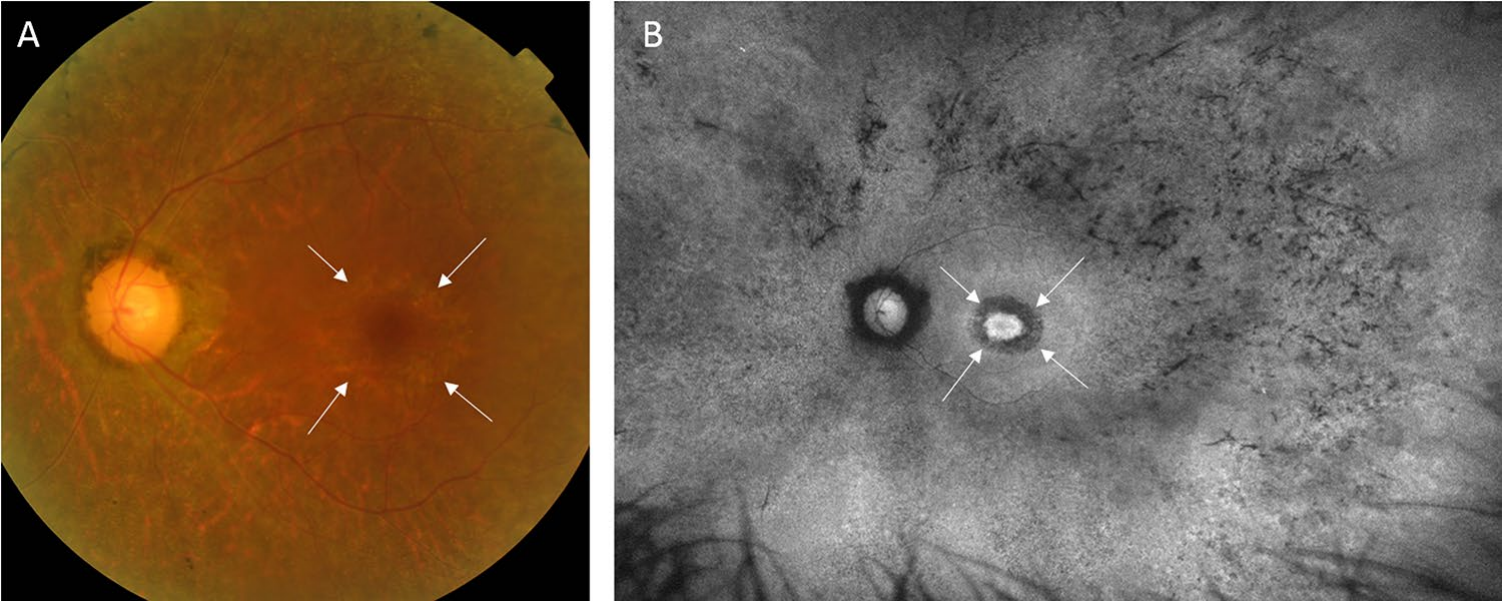
Representative images of a patient with Bull’s eye maculopathy. A 67-year-old woman with retinitis pigmentosa who preferred glasses that block middle-wavelength light. **(A)** A central normal red spot surrounded by a ring of atrophic pigment epithelium was observed on her fundus photograph. **(B)** The characteristic parafoveal atrophic ring was more apparent on autofluorescence images.

Next, we analyzed whether the FAF pattern is correlated with the types of photophobia. We found that all of patients with RP who preferred MWF glasses (7/47 patients; 14.9%) had Bull’s eye maculopathy, as seen on their fundus photographs and autofluorescence images (Fig. 2). (There was 100% agreement between the graders’ assessment of the Bull’s eye maculopathy in all 47 patients with RP.) Meanwhile, only 15% of patients who preferred SWF glasses (6/40 patients) had Bull’s eye maculopathy. The proportion of patients with bull’s eye maculopathy was significantly different between those who preferred MWF and SWF glasses (100%; 7/7 patients vs 15%; 6/40 patients) (p < 0.001). There were no other degenerative patterns on FAF related to the types of photophobia.

## Discussion

In this study, we investigated the primary source of photophobia in patients with IRD. We found that photophobia was induced primarily by short-wavelength light in patients with RP, whereas both short- and middle-wavelength light contributed to photophobia in patients with CRD. Additionally, middle-wavelength light also contributed to photophobia in RP patients with Bull’s eye maculopathy. These findings indicate that the mechanisms and the cells responsible for photophobia in IRD vary depending on the particular disease and the pathological condition of the patient.

Evaluating and investigating photophobia is challenging, because to date there is neither a unified clinical classification nor a general quantitative examination for the assessment of photophobia^5^. Nevertheless, photophobia expressed as a “white out” is generally attributed to diseases such as retinitis pigmentosa, glaucoma, diabetic retinopathy, etc. that are associated with the retina or optic nerve; diminished dynamic range due to change in retinal or nervous sensitivity is speculated to be the underlying mechanism for this symptom^17^. A “bright glare” occurs due to intraocular light scattering that is induced by disorders such as corneal diseases^19^ or cataract^20^. The “ocular pain” symptom (ocular pain and feeling that light of normal intensity is too intense) is specifically associated with ipRGCs and disorders like migraines which have trigeminal nerve-mediated mechanisms^21^. In the current study, almost all patients reported that their most severe symptom was a “white out”, followed by “ocular pain”; few patients complained of a “bright glare”. This indicates that photophobia in patients with IRD is less likely to occur due to light scattering and ipRGC-mediated mechanisms. The method established in this study can also be applied to photophobia in migraine, glaucoma and other ocular diseases.

In this study, photosensitive cells responding to short wavelengths including S-cones were indicated to be the primary source of photophobia in many patients with IRD, because SWF glasses attenuated the symptom. S-cones are the least numerous photoreceptors in the retina, accounting for only approximately 5 to 10% of all cone photoreceptor cells^9^. Although S-cones exist throughout the retina, they are present at a relatively higher density in the parafoveal region, despite their absence in the central fovea^22^. Thus, damage in this area would have a relatively larger impact on S-cones than on the other photoreceptors. Bull’s eye maculopathy is a term used to describe degeneration in this parafoveal area. It was initially described as a characteristic clinical appearance of chloroquine retinopathy in 1966^23^. In 1971, the term was used to illustrate a similar lesion in patients with IRD that is characterized by a central red spot surrounded by a ring of atrophied or mottled pigment epithelium^24^. Patients with Bull’s eye maculopathy often show blue-yellow defects; red-green defects begin to appear in more advanced stages^25^. This also supports the suggestion that Bull’s eye maculopathy is closely related to S-cone dominant damage. In this study, RP patients with Bull’s eye maculopathy preferred MWF glasses to SWF glasses. This was probably because they had less S-cones due to parafoveal degeneration and because the input from S-cones was already diminished; therefore, additional SWF glasses had little effect, whereas MWF glasses were effective.

Our study had some limitations. First, the sample size, particularly of the CRD group, was small. Further studies with larger sample sizes are required for a better understanding. Second, it was not possible to produce tinted glasses that distinguish between ipRGCs and rods, because the spectral sensitivities of these cells largely overlap. Thus, we were not able to identify the primary cell responsible for photophobia especially in patients who preferred MWF glasses. Third, we did not distinguish patients with yellow IOL from phakic patients. However, the preference of glasses and the improvement of symptoms were not different between phakic and pseudophakic patients. In fact, most pseudophakic patients (80.6%) preferred SWF glasses, even though yellow IOL was supposed to cut the short wavelength light. This indicate that yellow IOL was not a major factor. Finally, the evaluation of photophobia was essentially subjective; the questionnaire may not have thoroughly reflected the patient’s actual condition. Quantitative assessments such as palpebral aperture measurements in response to the light^26^ would be a way to reinforce our results.

In conclusion, the symptom of photophobia is primarily caused by short wavelength light in many patients with IRD, suggesting the involvement of S-cones. Particularly, parafoveal region with a high S-cone density seems to play a role in pathogenesis of photophobia. However, different wavelength of light causes photophobia depending on the disease and probably on the pathological condition and disease stage. Further studies are warranted to facilitate better patient care and a more profound understanding of the pathologic process of IRDs.
